# Ultrasonic Mucosectomy of the Gallbladder: A Histological Analysis

**DOI:** 10.1155/1993/31403

**Published:** 1993

**Authors:** Michel Gagner, Ramon Blanco, Ricardo L. Rossi

**Affiliations:** 1 Department of Surgery Hôtel-Dieu de Montréal 3840, St.-Urbain Montréal, Québec H2W 1T8 Canada; 2 Department of Surgery and Pathology Lahey Clinic Medical Center Burlington Mass. USA

## Abstract

The Cavitron Ultrasonic Surgical Aspirator (CUSA) may be used to remove mucosa of organs of the gastro-intestinal tract. A histological analysis was performed on gallbladders treated with a CUSA-mucosectomy to assess the extent and degree of mucosectomy and to evaluate parietal damage. The histological studies performed on three specimens of chronic cholecystitis revealed a complete mucosectomy except in areas where Rokitansky-Aschof sinuses were present. There was no evidence of parietal damage. The CUSA may be used to remove the mucosa of gallbladders without injury to other layers, and may have a potential application in procedures such as mucosal cholecystectomy.


*HPB Surgery*, 1993, Vol. 6, pp. 169–173
Reprints available directly from the publisher
Photocopying permitted by license only

© 1993 Harwood Academic Publishers GmbH
Printed in the United States of America

### ULTRASONIC MUCOSECTOMY OF THE GALLBLADDER

### A HISTOLOGICAL ANALYSIS

MICHEL GAGNER

*Department of Surgery, Hôtel-Dieu de Montréal, Montréal, Québec, Canada*

RAMON BLANCO and RICARDO L. ROSSI
*Department of Surgery and Pathology, Lahey Clinic Medical Center, Burlington, Mass., USA*

The Cavitron Ultrasonic Surgical Aspirator (CUSA) may be used to remove mucosa of organs of the gastro-intestinal tract. A histological analysis was performed on gallbladders treated with a CUSA-mucosectomy to assess the extent and degree of mucosectomy and to evaluate parietal damage. The histological studies performed on three specimens of chronic cholecystitis revealed a complete mucosectomy except in areas where Rokitansky-Aschof sinuses were present. There was no evidence of parietal damage. The CUSA may be used to remove the mucosa of gallbladders without injury to other layers, and may have a potential application in procedures such as mucosal cholecystectomy.

KEY WORDS: Cholecystitis, gallbladder, ultrasonic mucosectomy

### INTRODUCTION

Recently, different modalities have emerged in the treatment for benign gallbladder disease. Extracorporal shockwave lithotripsy can fragment cholelithiasis in selected patients and leaves a diseased gallbladder in place. Laparoscopic cholecystectomy has emerged as less traumatic than conventional cholecystectomy but should be performed in selected patients[1], requires a high degree of certain technical skills and has potentially disastrous complications[2]. Also, chemical cholecystectomy has been performed by laparoscopy[3]. Recurrence is usual after removal of gallstones,[4] whatever the method, when the gallbladder is left in place.

Bile composition and/or abnormal mucosa of the gallbladder are responsible for cholelithiasis. Removal of gallbladder mucosa could be therapeutic if the mucosa does not regenerate and if the cystic duct lumen is occluded. It is conceivable that a procedure could be designed to remove the mucosa of the gallbladder from the fundus by aspirating the lumen with subsequent scarring formation and obliteration of the lumen. This could be as therapeutic as complete removal of the gallbladder, without an increased risk of bile duct injury and vascular injury. Also, in cases

Address correspondence to: Michel Gagner, MD, Ph.D., Department of Surgery, Hôtel-Dieu de Montréal, 3840, St.-Urbain, Montréal, (Quebec) H2W 1T8 Canada where only a partial cholecystectomy is possible, the remnant tissue could have the mucosa removed. The Cavitron Ultrasonic Surgical Aspirator (CUSA) is a device which can easily aspirate and separate tissues like mucosa of the bowel[5]. We therefore performed an initial study on the tissues of gallbladders which were treated with the CUSA to assess mucosal integrity and to assess further damage on submucosal layers.

### PATIENTS AND METHODS

Three patients who underwent cholecystectomy for cholelithiasis and chronic cholecystitis had a portion of their gallbladder (1.5 <X> 3 cm) excised after the cholecystectomy. The specimen was transported in normal saline under sterile condition to the laboratory. Using the CUSA at maximal settings, the mucosa of half of the specimen was dissected/aspirated within 30 seconds, while the other half was used as control. The CUSA is a sterile probe connected to a power source that causes vibration along the longitudinal axis at an ultrasonic frequency of 23,000Hz. The vibrating tip is a hollow core of titanium connected to a suction apparatus. Saline is used to cool the tip which has a length of 6.4 cm and a diameter of 0.2 cm. After completion of the mucosectomy, the tissues were fixed in 10% formalin and processed in the standard fashion. The CUSA treated tissues and the control tissues were embedded separately in parafin. The sections were cut to a thickness of 3 mm and stained with hematoxylin and eosin. All histological sections were reviewed by a pathologist (R.B.).

### *Results*

Control sections showed intact epithelium and histopathological changes compatible with chronic cholecystitis in all three cases studied (Figure 1). The CUSA treated tissues showed an almost complete to complete loss of the glandular epithelium (Figure 2). In all three cases, residual glandular epithelium was focally present in the Rokitansky-Aschoff sinuses or diverticula (Figure 2). The parietal layers below the mucosa were not altered by the CUSA.

### *Comments*

The CUSA has multiple applications in surgery. The ultrasonic dissector allows a careful, and meticulous dissection of the liver parenchyma without damage of small vessels and bile ducts. It is specially useful for segmental and nonanatomical liver resection[6]. The CUSA is also used for subtotal removal of brain tumors[7], for cytoreduction in extensive ovarian cancer[8], to isolate and skeletonized vessels,[9,10] and has been used to clear the facial nerve from adjacent tissue[11]. However, CUSA use can also result in tissue damages to nearby structures. Temporary disruption of the perineurium of facial nerves before regeneration was evident in an animal model[11] and has resulted in nerve palsies in humans[12,13].

Hodgson *et al.* were the first to demonstrate the feasability of ultrasonic mucosectomy of the gastrointestinal tract. Mucosal proctectomy of the rectum has been successfully performed in five dogs, followed by endorectal pull-through of the ileum. The follow up studies revealed that the ileo-anal anastomosis had healed

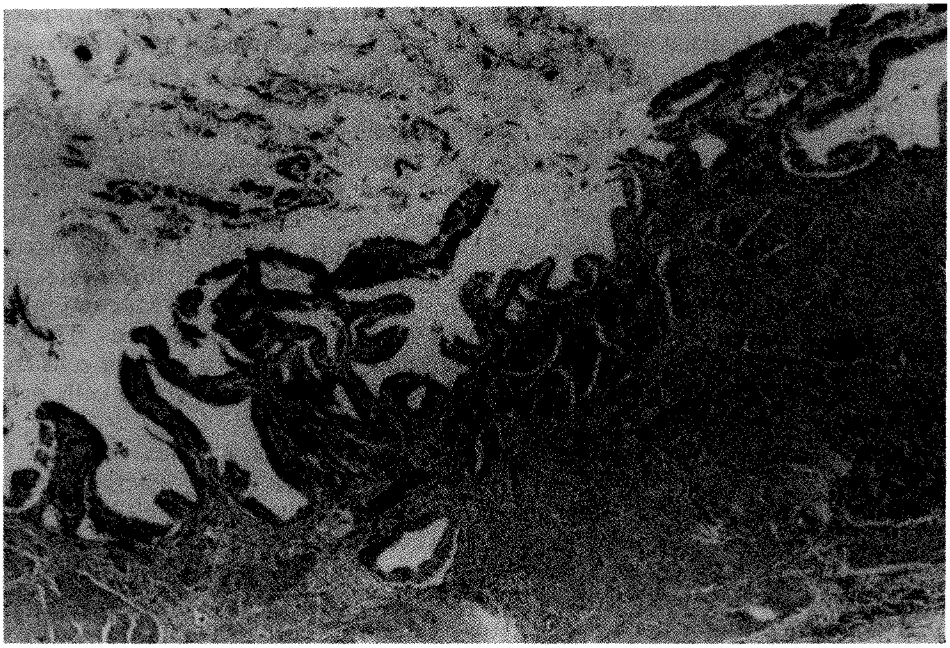

**Figure 1** Gallbladder histology. Normal mucosa before ultrasonic mucosectomy.

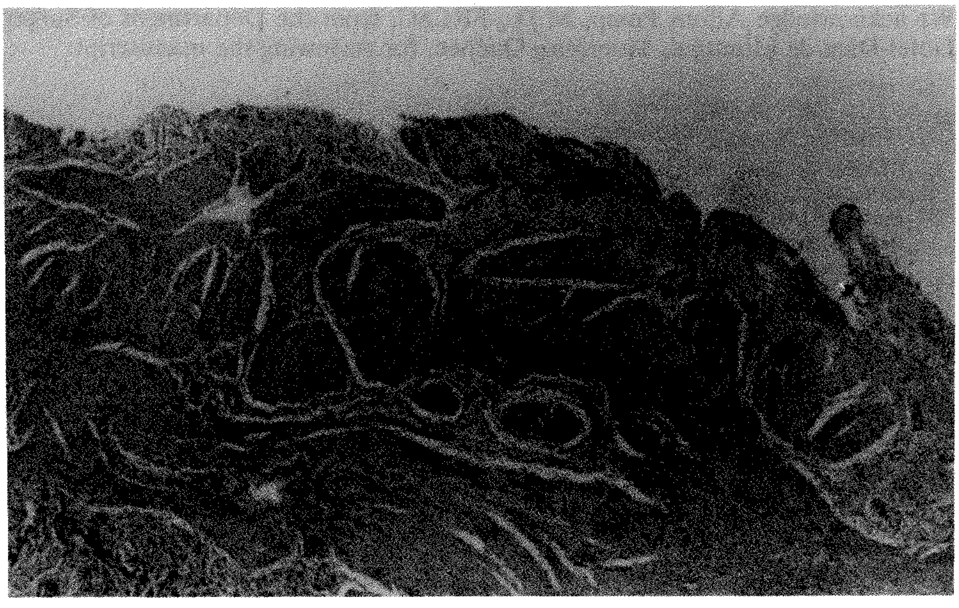

**Figure 2** Gallbladder histology. Absence of mucosa after ultrasonic aspiration.

perfectly without evidence of stricture formation[14]. Histological analysis revealed that not only the mucosa but also the muscularis mucosa were destroyed. Some portions of the submucosa also showed signs of damages as evidenced by hemorrhage, edema and partial necrosis. Histological studies performed two months later revealed no mucosa, a thinner and fibrous submucosa and an intact muscularis propria.

Removal of glandular epithelium was almost complete in the three cases studied, and isolated islands of glandular epithelium remained only in Rokitansky-Aschoff sinuses. It seems possible that these residual islands of epithelium would not have persisted in normally distended gallbladders, since it is well known that folds disappear when the gallbladder is distended. It is possible that in removed fragments of gallbladder wall the contraction of the smooth muscle fibers present in fibromuscular layer could have produced infolding of the mucosa. Mucosal infolding might have prevented the total removal of epithelium in the sinuses. The cells aspirated by the CUSA can be viable. Aspirated Lewis lung carcinoma cells from female mice grew *in vitro* and *in vivo*[15]. Therefore spillage of viable cells in the peritoneal cavity is a potential problem.

Partial cholecystectomy can be performed in high-risk patients or when the inflammatory process has distorted the anatomy to a point where a formal cholecystectomy would be risky[16]. The cauterization of the mucosa left behind after the partial cholecystectomy is thought to prevent the occurrence of infection and gallbladder carcinoma[16]. It is uncommon to encounter such complications following partial cholecystectomy. CUSA mycosectomy thus appears to have potential therapeutic applications in selected cases.

### *Acknowledgement*

We acknowledge Alfons Pomp, M.D., FRCSC, from the Department of Surgery Hôtel-Dieu de Montréal, Montréal, Québec, for reviewing the manuscript.

### *References*

1. Dubois, F., Icard, P., Berthelot, G. and Levard, H. (1990) Coelioscopic cholecystectomy. *Ann.Surg.* **211** (1), 60–62
2. Zucker, K.A., Mailey, R.W., Gadacz, T.R. and Imbembo, A.L. (1990) Laparoscopic cholecystectomy: A plea for cautious enthusiasm (abstract). Society for Surgery of the alimentary tract. San Antonio, Tx, May 15-16
3. Cuschieri, A., Abd El Ghany, A.A.B. and Holley, M.P. (1989) Successful chemical cholecystectomy: A laparoscopic guided technique. *Gut*, **30**, 1786–1794
4. Gibney, R.G., Chow, K., Benny, So C., Rowley, V.A., Cooperberg, P.L. and Burhenne, H.J. (1989) Gallstone recurrence after cholecystolithotomy. *AJR*, **153**, 287–289
5. Hodgson, W.J.B., Finkelstein, J.I., Woodriffe, P. and Aufses, A.H. Jr. (1979) Continent anal ileostomy with mucosal proctectomy: A bloodless technique using a surgical ultrasonic aspirator in dogs. *Br.J.Surg.*, **66**, 857–860
6. Putnam, C.W. (1983) Techniques of ultrasonic dissection in resection of the liver. *Surg.Gynecol.Obstet.*, **157** (5), 474--478
7. Albright, A.L. and Sclabassi, R.J. (1985) Use of The Cavitron ultrasonic surgical aspirator and evoked potentials for the treatment of thalamic and brain stem tumors in children. *Neurosurgery*, **17**(4)L, 564–568
8. Adelson, M.D., Baggish, M.S., Seifer, D.B., Cassell, S.L. and Thompson, M.A. (1988) Cytoreduction of ovarian cancer with the Cavitron ultrasonic surgical aspirator. *Obstet.Gynecol.*, **71**(1), 140--143

9. Melzer, R.B., Wood, T.W., Landau, S.T. and Smith, J.A., Jr. (1985) Combination of CUSA and neodymium: YAG laser for canine partial nephrectomy. *J. Urol.*, **134**(3), 620–622
10. Suma, H., Fukumoto, H. and Takeuchi, A. (1987) Application of ultrasonic aspirator for dissection of the internal mammary artery in coronary artery bypass grafting. *Ann. Thorac. Surg.*, **43**(6), 676–677
11. Gleeson, M.J. and Felix, H. (1986) The cavitron ultrasonic surgical aspirator system. An anatomical and physiological study of the effect of its use on the rat facial nerve. *Clin. Otolaryngol.*, **11**(3), 177–187
12. Gleeson, M.J., Fisch, U. and Felix, H. (1987) A morphological study of the effect of the Cavitron ultrasonic surgical aspirator system near human peripheral nerves. *Arch. Otolaryngol. Head Neck Surg.*, **113**(5), 530–532
13. Ridderheim, P.A., von Essen, C. and Zetterlund, B. (1987) Indirect injury to cranial nerves after surgery with Cavitron Ultrasonic Surgical Aspirator (CUSA). Case Report. *Acta Neurochir.*, (Wien), **89**(1-2), 84–86
14. Heimann, T.M., Kurtz, R.J., Shen, S. and Aufses, H.A. Jr. (1984) Mucosal protectomy using an ultrasonic scalpel. *Am. J. Surg.*, **147**(6), 803–806
15. Oosterhuis, J.W., Lung, P.F., Verschueren, R.C. and Oldhoff, J. (1985) Viability of tumor cells in the irrigation fluid of the cavitron ultrasonic surgical aspirator CUSA after tumor fragmentation. *Cancer*, **56**(2), 368–370
16. Bickel, A., Lunsky, I., Mizrahi, S. and Stamler, B. (1990) Modified subtotal cholecystectomy for high-risk patients. *Can. J. Surg.*, **33**(1), 13–14

*(Accepted by S. Bengmark 15 June 1992)*

